# Plant Mitochondrial Inner Membrane Protein Insertion

**DOI:** 10.3390/ijms19020641

**Published:** 2018-02-24

**Authors:** Renuka Kolli, Jürgen Soll, Chris Carrie

**Affiliations:** 1 Department of Biology I, Botany, Ludwig-Maximilians-Universität München, Großhaderner Strasse 2-4, D-82152 Planegg-Martinsried, Germany; renuka.kolli@biologie.uni-muenchen.de (R.K.); soll@lmu.de (J.S.); 2 Munich Center for Integrated Protein Science, CiPSM, Ludwig-Maximilians-Universität München, Feodor-Lynen-Strasse 25, D-81377 Munich, Germany

**Keywords:** plant mitochondria, membrane insertion, Oxa, twin-arginine translocation

## Abstract

During the biogenesis of the mitochondrial inner membrane, most nuclear-encoded inner membrane proteins are laterally released into the membrane by the TIM23 and the TIM22 machinery during their import into mitochondria. A subset of nuclear-encoded mitochondrial inner membrane proteins and all the mitochondrial-encoded inner membrane proteins use the Oxa machinery—which is evolutionarily conserved from the endosymbiotic bacterial ancestor of mitochondria—for membrane insertion. Compared to the mitochondria from other eukaryotes, plant mitochondria have several unique features, such as a larger genome and a branched electron transport pathway, and are also involved in additional cellular functions such as photorespiration and stress perception. This review focuses on the unique aspects of plant mitochondrial inner membrane protein insertion machinery, which differs from that in yeast and humans, and includes a case study on the biogenesis of Cox2 in yeast, humans, two plant species, and an algal species to highlight lineage-specific similarities and differences. Interestingly, unlike mitochondria of other eukaryotes but similar to bacteria and chloroplasts, plant mitochondria appear to use the Tat machinery for membrane insertion of the Rieske Fe/S protein.

## 1. Introduction

Mitochondria are essential organelles found in all eukaryotic cells. They are popularly known as the “powerhouse of the cell” for generating more than 90% of the cellular energy in the form of ATP. Additionally, they perform several vital cellular functions that include the synthesis of heme and iron-sulfur clusters, lipid metabolism, maintenance of calcium homeostasis, thermogenesis, innate immunity, and the activation of apoptosis [1]. Approximately 2 billion years ago, the ancestors of mitochondria were once free-living prokaryotes. The primordial eukaryote most likely arose from the symbiosis between a facultative anaerobic archaeon (host) and an α-proteobacterium (mitochondrial ancestor) [2]. Gradually over time, this single unicellular primitive eukaryote multiplied and evolved to form the wide range of complex multicellular life that we see today. Due to the monophyletic origin of mitochondria [3], the fundamental mitochondrial features and functions are conserved, despite the lineage-specific differences. Compared to mitochondria of other lineages, plant mitochondria have evolved several unique features: Plant mitochondrial genomes are larger and highly variable in size [4]. Their transcripts undergo extensive processing and editing [5,6]. They have a branched electron transport chain and the composition of the respiratory complexes is slightly different [7]. Moreover, plant mitochondria are involved in photorespiration and have evolved to coexist with chloroplasts, a second endosymbiotically-derived organelle.

The mitochondrial inner membrane (MIM) is extensively folded into cristae, which vastly increases the surface area [8]. Thus, the MIM is able to house more than 50% of the total mitochondrial proteins that are crucial for numerous aspects of mitochondrial functions, including oxidative phosphorylation, protein import, metabolite transport, maintenance of mitochondrial morphology, and nucleoid segregation [9,10]. Having descended from eubacterial ancestors, the MIM proteome is well-conserved across eukaryotic species; however, minor differences do occur [11]. A recent yeast mitochondrial proteomics approach assigned 245 integral and 236 peripheral MIM proteins that together represent 59% of the total mitochondrial proteome [12]. The protein composition of the plant MIM is likely to be roughly similar [13]. Respiratory complexes comprise 80% of the MIM proteins and are preferentially located in cristae membranes, whereas protein translocation complexes and carrier proteins are enriched in the inner boundary membrane [9]. The respiratory complexes I to V are involved in the main mitochondrial function of cellular energy generation. All of these complexes except for complex II are made up of a mosaic of both nuclear- and mitochondrial-encoded proteins. The respiratory complexes are not randomly distributed in the MIM but assemble into supramolecular structures [14,15,16,17]. While I_1_ + III_2_ is the most abundant supercomplex of plant mitochondria, other supercomplexes—I_2_ + III_4_, III_2_ + IV_(1–2)_, I_1_ + III_2_ + IV_(1–4)_, and V_2_—are found in lower abundance [18]. The MICOS (the mitochondrial contact site and cristae organizing system) complex, complex V dimer, and cardiolipin are crucial for cristae formation in the MIM [19,20,21].

Coordinated gene expression, insertion, and assembly of MIM proteins are essential for the survival of eukaryotic cells. This review provides an overview of presently known machineries and mechanisms for the insertion of proteins into plant MIM, with a particular focus on insertion from the matrix side. A case study comparing the biogenesis of Cox2 in yeast, humans, and three different plant species not only highlights some differences observed in plant mitochondria but also indicates that our understanding of biogenesis of plant MIM proteins is presently incomplete.

## 2. Mitochondrial Protein Import Machinery

**Figure 1:**
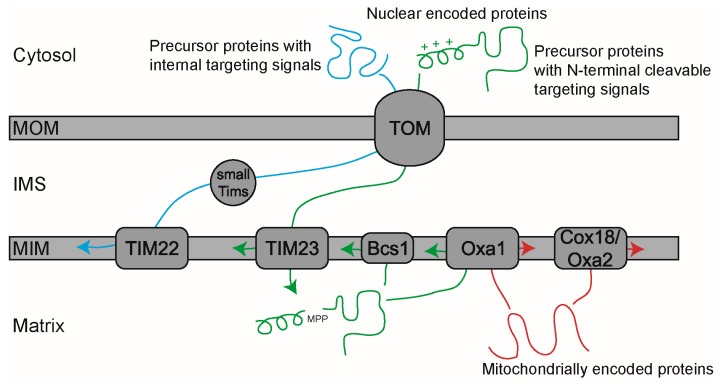
Mitochondrial protein transport and inner membrane insertion pathways. The MIM proteins can follow several routes for membrane insertion. There are the two translocase complexes in the inner membrane, TIM22 and TIM23, which laterally insert nuclear-encoded proteins into the MIM. Proteins with internal targeting signals are inserted into MIM by TIM22, while those with cleavable N-terminal targeting signals are inserted by TIM23. The conservatively sorted proteins are first targeted to the matrix by TIM23 and then inserted to the MIM in a manner reminiscent of bacterial inner membrane protein insertion. These conservatively sorted and mitochondrial-encoded proteins require Oxa1 or Cox18 (Oxa2) for membrane insertion. The newest member for conservative sorting is the Bcs1 protein for membrane insertion of the Rieske Fe/S protein. MOM = mitochondrial outer membrane, IMS = intermembrane space, MIM = mitochondrial inner membrane, MPP = mitochondrial processing peptidase, Oxa = oxidase assembly factor, TIM = translocase of the inner membrane.

During the course of eukaryotic evolution, most of the genes that were originally present in the genome of the α-proteobacterial endosymbiotic ancestor of mitochondria were gradually transferred to the host nucleus with only a very small number being retained in the genome of the mitochondria [22]. Consequently, the basic mitochondrial protein import machinery consisting of the core subunits had developed even before the divergence of eukaryotes into fungi, plants, and metazoans [23]. Later on, during the evolution of each species, a number of lineage-specific subunits were added while others were lost. Today, the vast majority of mitochondrial proteins are synthesized as precursors in the cytosol and are targeted to mitochondria via N-terminal mitochondrial-targeting sequences (Figure 1). At the mitochondrial outer membrane, the precursor proteins interact with receptor subunits of the translocase of the outer membrane (TOM) complex [24]. The TOM complex contains an aqueous pore through which all the nuclear-encoded mitochondrial proteins must pass in order to cross the outer membrane. After emerging from the TOM complex, the mitochondrial-targeting sequences interact with the TIM23 (translocase of the inner membrane) complex, which mediates the translocation of the precursor proteins across the inner membrane into the matrix in a membrane potential- and ATP-dependent manner [25,26,27] (Figure 1). In the matrix, the targeting sequences are proteolytically removed and the proteins are folded into their respective native structures [28]. For a more detailed description of the mitochondrial protein import machinery of yeast [29,30], humans [31,32], and plants [33], interested readers may refer to the recent reviews. While the majority of protein import components are conserved across lineages, in plants, the gene family members encoding the protein import components have expanded. For example, while yeast contain a single gene encoding each of Tim17, Tim23, and Tim22, all three of which belong to the PRAT (preprotein and amino acid transporters) family, the *Arabidopsis* genome contains 17 genes encoding different PRAT family members, of which ten are located in mitochondria, six in chloroplasts, and one is dual targeted [33].

## 3. Membrane Insertion of the MIM Proteins Synthesized in the Cytosol

Whereas all soluble matrix-localized proteins follow the route outlined above in the mitochondria of different organisms, MIM proteins have several different targeting and import pathways. First, the MIM proteins possessing N-terminal-targeting signals are arrested at the TIM23 complex, followed by lateral release into the membrane (Figure 1) [34]. So far, all proteins identified to be using this pathway contain only a single membrane-spanning transmembrane helix (TMH) [35]. The pore-forming subunit Tim23, its paralog Tim17, and the presequence receptor Tim50 form the essential catalytic core of the TIM23 complex [36]. A plant-specific C-terminal extension of *Arabidopsis* Tim17-2 is in contact with the outer membrane and is essential for protein import [37]. In yeast, the N-terminal extension of Tim23 links the inner and the outer membranes; however, all three isoforms of *Arabidopsis* Tim23 lack this domain [38]. Apart from being the subunits of TIM23, Tim23-2 and B14.7 are also present as part of the respiratory complex I and an inverse relationship between their abundance in the two complexes was shown to coordinate mitochondrial activity and biogenesis [39]. This appears to be a plant-specific mechanism. Interestingly, the presequence degrading peptidase of plants is dual targeted to both mitochondria and plastids to degrade mitochondrial-targeting sequences, as well as plastid-targeting signals [40,41].

The second group of MIM proteins can be inserted by the second TIM translocase, TIM22, also known as the carrier translocase as the majority of its substrates belong to the mitochondrial carrier protein family [42]. Carrier proteins are polytopic membrane proteins containing multiple hydrophobic internal signals and generally lacking N-terminal cleavable targeting signals [35]. They are transferred from TOM to the TIM22 via the heterohexameric chaperone complex that is composed of small Tim proteins in the intermembrane space (IMS), which prevents aggregation or misfolding of the hydrophobic precursors [42] (Figure 1). In yeast, the TIM22 complex consists of the pore-forming Tim22 protein, Tim54 receptor, Sdh3, and Tim18 [43]. Since no plant homologs have been found for Tim54, Tim18, and Tim12 (a small Tim protein), which are essential for carrier import in yeast [44], it is highly likely that novel plant proteins perform similar functions. These additional components are yet to be identified. Upon activation of Tim22 by the membrane potential, substrate proteins are laterally released into the MIM (Figure 1). An exception to the general rule is that numerous plant carrier proteins possess a cleavable N-terminal-targeting sequence, despite displaying the typical tripartite structure and being homologous to other members of the carrier family [45]. These extensions appear to be non-essential for correctly targeting to the mitochondria. Nevertheless, they might be important for enhancing the import specificity and efficiency and might avoid mistargeting to chloroplasts [46]. After or during import, the extension is removed in a two-step process: the first processing by MPP (mitochondrial processing peptidase) and the second processing by a putative serine protease in the IMS [47].

The third group of MIM proteins displays a slightly more complicated sorting process. Firstly, they are translocated into the matrix and then integrated into the MIM from the matrix side of the membrane (Figure 1). This pathway is called the conservative sorting pathway because the direction of insertion is the same as that observed for bacterial membrane proteins [48]. While not just displaying a conserved direction, several aspects of this pathway also appear to be similar to that of bacteria, including the dependence on the membrane potential for insertion and adherence to the positive-inside rule for the charge distribution flanking the TMHs [49]. Since conservatively sorted proteins share similar pathways for membrane insertion as the mitochondrially encoded proteins, they will be discussed together in detail.

## 4. Membrane Insertion of MIM Proteins from the Matrix Side

### 4.1. Membrane Insertion of MIM Proteins Synthesized in the Matrix

**Figure 2:**
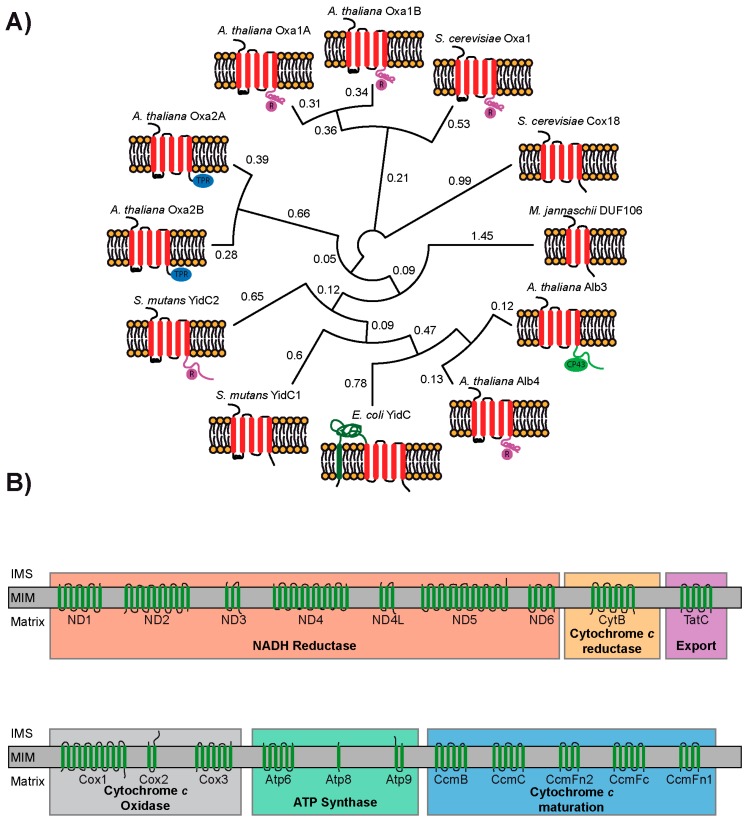
Phylogenetic and structural analysis of the YidC/Oxa1/Alb3 family and the mitochondrial-encoded membrane proteins of *Arabidopsis thaliana*. (**A**) A phylogenetic tree was generated by Clustal omega based on the full-length protein sequences for the indicated species of the YidC/Oxa1/Alb3 family [60]. The numbers next to each node represent the measure of support for the node. The conserved five TMH secondary structures are found in all members (except archaea) of the protein family. Differences at the C-terminal ends are indicated by different colors: pink = ribosome-binding, blue = TPR domain, green = CP43-interacting. (**B**) Transmembrane topologies of the 20 putative MIM proteins encoded in the mitochondrial genome of *Arabidopsis thaliana*. The proteins are displayed in an N- to C-terminus orientation going from left to right.

The most central protein in the biogenesis of mitochondrial inner membrane proteins is Oxa1 (oxidase assembly factor 1) as it has been demonstrated to play critical roles in both co- and post-translational integration of MIM proteins [50]. Oxa1 was first identified in 1994 in a yeast mutant that failed to assemble cytochrome c oxidase (COX), resulting in a respiration-deficient phenotype [51,52]. Subsequent work has identified that Oxa1 is part of a larger family of proteins called the YidC/Oxa1/Alb3 family. The YidC/Oxa1/Alb3 family of membrane proteins plays a key role in the biogenesis of the respiratory chain complexes in bacteria (YidC) and mitochondria (Oxa1) and in the assembly of photosynthetic complexes in chloroplasts (Alb3). They insert proteins into membranes and help in protein folding and complex assembly [53,54]. Very recently, three members of this family—Get1 (guided entry of tail-anchored protein 1), EMC3 (Endoplasmic reticulum membrane complex 3), and TMCO1 (transmembrane and coiled-coil domain-containing protein 1)—were identified to play roles in membrane insertion in the endoplasmic reticulum as well [55]. The conserved hydrophobic core domain consisting of five TMHs, which catalyzes membrane protein insertion, is present in each family member (Figure 2A). This domain can be functionally exchanged among different members of the protein family [56]. However, the soluble N- and C-terminal domains are variable and sometimes perform specialized functions (Figure 2A). Co-translational membrane insertion of substrate proteins is supported by the C-terminal ribosome-binding domain of the mitochondrial Oxa1, [57,58] whereas the cpSRP43-binding region at the C-terminus of the chloroplast Alb3 is crucial for targeting LHCPs (light-harvesting chlorophyll-binding proteins) to the thylakoid membrane [59] (Figure 2A).

Usually, all organisms possess at least two homologs of the YidC/Oxa1/Alb3 family, performing non-redundant functions. Exceptionally, the Gram-negative bacteria have only a single YidC protein with an extra TMH0 near the N-terminus, an extended periplasmic loop between TMH0 and TMH1, and a unique cytoplasmic coiled-coil region between TMH1 and TMH2 [61] (*E. coli* YidC in Figure 2A). YidC catalyzes the membrane insertion of several substrate proteins with one or two TMHs and lacking highly charged hydrophilic domains in the periplasmic side. YidC undergoes a conformational change upon co-translational substrate insertion [62]. The recent X-ray structures of YidC from *B. halodurans* and *E. coli* revealed a hydrophilic groove, which is open from the cytoplasm and the lipid bilayer but is closed from the periplasmic side. Moreover, the relatively shorter TMH3, 4, and 5 cause membrane thinning around YidC. Upon interaction with YidC in the groove region, the TMHs of the substrate slide into the lipid bilayer [63,64]. It is currently hypothesized that the mechanism of protein insertion by Oxa and Alb proteins is similar to YidC since they can be modeled on the known structure of YidC. YidC not only acts independently but also cooperates with SecYEG to facilitate the correct membrane integration and folding of membrane proteins [65]. Recently, it was shown that YidC contacts the interior of the SecY channel to form a ligand-activated and voltage-dependent complex [66]. Upon substrate binding, it facilitates the partitioning of nascent membrane proteins into the lipid environment by reducing the hydrophobicity of the SecY lateral gate [66]. Most Gram-positive bacteria have two YidC paralogs. In *Streptococcus mutans*, YidC2 has a ribosome-binding domain while YidC1 lacks it [67] (Figure 2A). The substrate profile of the bacterial YidC appears to be much larger than that of its organellar counterparts, Oxa1 and Alb3. These include the subunits a, b, and c of ATP synthase, TssL (a tail-anchored protein), and CyoA (cytochrome *bo*_3_ oxidase subunit 2). With LacY (lactose permease) and MalF (maltose transporter), YidC also functions as a chaperone to assist in the folding and stability of the nascent polypeptide [68,69]. Similar to the bacterial YidC, the chloroplast Alb3 may also cooperate with cpSecY to perform co-translational membrane insertion of proteins into the thylakoid membrane [70], apart from its role in the post-translational insertion of LHCPs [71]. Its paralog Alb4 is involved in the assembly and stability of ATP synthase [72,73]. In *Chlamydomonas reinhardtii*, while the insertion of D1, a core subunit of photosystem II (PSII), is mediated by cpSecY, its assembly into PSII is strictly dependent on Alb3.1 [74]. Moreover, its paralog Alb3.2 is also exclusively involved in the assembly of PSI and II [75].

In mitochondria, Oxa1 is involved in the co-translational membrane insertion of polytopic membrane proteins, such as Cox2, into the MIM [57]. In cooperation with a peripheral membrane protein, Mba1 (multi-copy bypass of AFG3), the positively charged coiled-coil domain at the Oxa1 C-terminus interacts with the negatively charged 21S RNA of the large subunit of the ribosome, which is in proximity to the polypeptide exit tunnel [76,77] (Figure 2A). Thus, Oxa1 is able to directly contact the nascent polypeptide very early during the translation and inserts it efficiently into the membrane. The matrix-exposed loop between the TMHs 1 and 2 may also contribute to ribosome binding. The Oxa1–ribosome interaction not only promotes co-translational insertion but is also critical for the assembly of the COX subunits [78]. Similarly, during biogenesis of the F_o_ part of ATP synthase, although Oxa1 is not involved in the insertion of Atp9 into the MIM, it is required for the assembly of Atp9 with Atp6 [79]. Thus, Oxa1 can also function in a chaperone-like manner in addition to performing membrane insertion. While Oxa1 is conserved and Oxa1 proteins from different organisms are exchangeable, their specific functions can differ slightly: The yeast Oxa1 is involved in the biogenesis of complexes IV and V, whereas the human Oxa1 is required for the biogenesis of complexes I and V [50,80,81]. In contrast, Oxa1 depletion affects the levels of complexes I and IV in *Neurospora crassa* [82]. The paralog of Oxa1, Cox18 (Oxa2), lacks the ribosome-binding coiled-coil domain. Hence, with respect to the presence or absence of the ribosome-binding domain, the mitochondrial Oxa1 and Cox18 resemble the Gram-positive YidC2 and YidC1, respectively [67] (Figure 2A). Nevertheless, unlike other family members with several known substrate proteins, for Cox18, Cox2 is the only known substrate [83]. Cox18 performs a post-translational role in efficient topogenesis and stability of Cox2 during the assembly of COX.

In contrast to yeast and humans, due to independent gene duplications, there are four Oxa proteins in *Arabidopsis thaliana*: Oxa1a, Oxa1b, Oxa2a, and Oxa2b. Oxa1a and Oxa1b possess a coiled-coil domain similar to yeast Oxa1 (Figure 2A) [84]. Interestingly, Oxa2a and Oxa2b possess a tetratricopeptide repeat (TPR) domain consisting of four TPR motifs near the C-terminus, which is a plant-specific feature and is not found in any other known members of the protein family [85] (Figure 2A). TPR domains are involved in protein–protein interactions in a variety of cellular proteins. For example, the TPR domain of the TOM receptor, Tom70, interacts with the cytosolic chaperones Hsc70/Hsp70 and Hsp90, which guide precursor proteins [86]. This interaction is crucial for mitochondrial precursor targeting and translocation. Along these lines, it can be speculated that the TPR domains of Oxa2a and Oxa2b perhaps bind the matrix chaperones for facilitating the membrane protein insertion and/or help in complex assembly. Except for Oxa1b, the other three Oxa proteins are essential genes in *Arabidopsis*. This indicates that Oxa1a, Oxa2a, and Oxa2b play vital non-redundant roles in MIM protein biogenesis in all stages of plant development, while Oxa1b is probably not so important for normal plant growth and physiology [84].

In terms of plant Oxa proteins, two questions are immediately obvious: The first is why do plants have four Oxa proteins when most other organisms including humans and yeast have only two? Secondly, what is the role of the TPR domains found in the plant Oxa2 proteins? The answer to the first question may be just that plant mitochondria contain many different substrates compared to yeast and humans. This idea is supported by the fact that many plant mitochondrial genomes encode for more membrane proteins than those of yeast and humans [87]. For example, the *Arabidopsis* mitochondrial genome encodes for 20 putative membrane proteins displaying a wide range of membrane topologies (Figure 2B), whereas yeast and human mitochondrial genomes encode seven and thirteen membrane proteins, respectively. Although the major plant respiratory chain protein complexes are fairly well characterized and structurally resemble their counterparts in fungi and mammals, the electron transport chain of plant mitochondria differs significantly due to the presence of several plant-specific alternative oxidoreductases such as alternative oxidase (AOX) and rotenone-insensitive NAD(P)H dehydrogenases [88,89]. Furthermore, some of the respiratory complexes of plant mitochondria have numerous extra subunits that are not found in those of bacteria, fungi, and animals. To date, the functions of only some of them have been revealed. Complex I of plants is especially large, comprising of approximately 50 subunits [90]. In contrast to complex I of bacteria or other eukaryotic lineages, plant complex I has an additional spherical domain attached to the membrane arm on the matrix side, which includes five carbonic anhydrase-like proteins [91,92]. It was recently shown that these proteins are essential for complex I assembly and specifically influence central mitochondrial metabolism [93]. Nine more plant-specific subunits of complex I identified need further investigation. Complex II of flowering plants includes four additional subunits termed Sdh5, Sdh6, Sdh7, and Sdh8 [94]. While Sdh6 and Sdh7 represent the missing segments of plant Sdh3 and Sdh4, respectively, the functions of Sdh5 and Sdh8 are currently unknown [95]. Plant complex IV probably contains at least six additional putative plant-specific subunits that need further confirmation [94]. Similarly, plant complex V also includes several plant-specific subunits [96]. Any of the abovementioned plant-specific proteins of the respiratory chain could require either more diverse Oxa proteins or possibly require the plant-specific TPR domain for proper sorting and insertion.

In trying to answer the second question above, a specific role for the TPR domains found in Oxa2a and Oxa2b is hard to predict as these are the only known YidC/Oxa1/Alb3 family proteins to contain a TPR domain. As stated before, it is possible that they could interact with the chaperones of the matrix; however, since the mitochondrial Hsp70 lacks the classic EEVD motif required for TPR interactions, this hypothesis is weakened [97]. So far, in yeast and humans the only known substrate for Oxa2 (Cox18) is Cox2. More specifically, Oxa2 is required for the insertion of the second TMH of Cox2 and translocation of the C-terminus from the matrix to the IMS [83]. It is highly likely that either Oxa2a or Oxa2b or both of them play a similar role in plants. However, since both are essential, it is unlikely they have overlapping functions. The role of the TPR domain in Cox2 biogenesis could be similar to that of Mss2 (mitochondrial splicing system-related protein), which is a membrane-associated TPR-containing protein that cooperates with Cox18 during Cox2 biogenesis in yeast [98]. It is possible that plants have just formed a single protein of Cox18 and Mss2 via gene fusion. However, there is no significant similarity between the TPR domains of Oxa2 proteins and Mss2. Interestingly, no TPR protein has to date been found to be required for human Cox2 biogenesis. Considering that either Oxa2a or Oxa2b is possibly required for Cox2 biogenesis, then what is the other doing? Only future functional studies will be able to answer this question.

### 4.2. Membrane Insertion of MIM Proteins Imported into the Matrix

#### 4.2.1. Oxa-Dependent Insertion

Oxa1 functions not only in the insertion of mitochondrial-encoded proteins but also in promoting the proper insertion of a variety of nuclear-encoded proteins containing multiple TMHs. While the role of Oxa1 in the insertion of mitochondrial-encoded proteins has been extensively studied, more recently there has been a focus on its role in the conservative sorting of nuclear-encoded MIM proteins. For the MIM insertion of nuclear-encoded proteins with a complex membrane topology, TIM23 and Oxa1 have to cooperate [99]. After translocation into the matrix via TIM23, some precursor proteins are inserted into the MIM with the help of Oxa1. Interestingly, many of the proteins sorted in this manner have bacterial homologs [48,100]. Oxa1 is required for its own biogenesis based on this mechanism [101]. In other cases, only some TMHs of the precursor proteins are laterally sorted by TIM23 while the remaining are first translocated into the matrix and then exported into the membrane by Oxa1. For instance, during the biogenesis of Mdl1, TIM23 laterally inserts the first pair of TMHs into the MIM while the subsequent pair is translocated into the matrix by TIM23 for MIM insertion by Oxa1 [102]. Generally, laterally inserted TMHs are more hydrophobic than those that are first translocated into the matrix. Another example is the biogenesis of Sdh4 in yeast. Sdh4 contains three TMHs, the first two are translocated through TIM23 into the matrix and exported by Oxa1, whereas the third is arrested in the TIM23 complex and laterally inserted into the MIM [103]. Interestingly, Sdh4 of flowering plants is truncated and only contains the third TMH found in yeast [104]. However, the additional subunit Sdh7 resembles the missing part and contains the two missing TMHs [95]. Thus, it can be speculated that plants simplified the complicated insertion process of Sdh4 by splitting it into two parts so that one part can follow the TIM23-dependent Oxa1 insertion while the other can be laterally inserted by TIM23. Since the two subunits of the carrier translocase Sdh3 and Tim18 also use the TIM23-Oxa1 pathway, depletion of Oxa1 leads to defects in the biogenesis of numerous carrier proteins, apart from a broad spectrum of other MIM proteins [105].

#### 4.2.2. Oxa-Independent Insertion Using Bcs1 and Tat

The most recent sorting pathway to be identified in mitochondria is the Bcs1 pathway for the insertion of the Rieske Fe/S protein of complex III into the inner membrane [106,107]. The Rieske Fe/S protein undergoes a unique maturation and membrane insertion pathway and at this time is the only protein known to use this route. Similar to many other nuclear-encoded mitochondrial proteins, the Rieske Fe/S protein contains a cleavable N-terminal-targeting peptide and is translocated across the outer membrane through the TOM complex and through the inner membrane via the TIM23 complex [107]. Even though the Rieske Fe/S protein is a membrane protein, it is fully translocated into the matrix and during its maturation has a soluble intermediate [108]. Once inside the matrix, the N-terminal-targeting signal is cleaved off in two steps [108]. Then, the C-terminus is folded and the Fe/S cluster is inserted [106]. After C-terminal folding, the Rieske Fe/S protein is inserted into the MIM by Bcs1 (cytochrome *bc*_1_ synthesis) so that it can reach its final topology of N-in and C-out [106]. Therefore, uniquely for mitochondria, the previously folded C-terminus of the Rieske Fe/S protein must be translocated back through the inner membrane into the IMS. This is in stark contrast to other conservative pathways where substrates remain in an unfolded state during membrane insertion.

Bcs1 is an AAA-type ATPase protein and has been demonstrated to be the only protein required to fully translocate the folded C-terminus of the Rieske Fe/S protein across the MIM. While this may be true in yeast and mammalian mitochondria, it has recently been demonstrated that the closest related protein in plants is located in the outer membrane—not the inner membrane—and plays no role in complex III biogenesis [109]. More recent work has sought to determine if plant mitochondria use a different pathway for Rieske Fe/S membrane insertion. Here, it could be demonstrated that plant mitochondria most likely use a twin-arginine translocation (Tat) pathway in place of the Bcs1 pathway [110]. This is interesting because in bacteria and chloroplasts a Tat pathway is also utilized for the insertion of Rieske Fe/S proteins into the cytochrome *bc*_1_ complex and the cytochrome *b*_6_*f* complex, respectively [111]. The evidence for plant mitochondria using a Tat pathway are that plant mitochondria contain two subunits of a potential Tat pathway (TatB and TatC) and that modification of the predicted Tat signal within the plant Rieske Fe/S protein blocks the assembly into complex III [110]. It was further shown that knockouts of the mitochondrial TatB were lethal in *Arabidopsis*. However, several questions still remain unanswered: First, where is the mitochondrial TatA? The majority of Tat pathways require TatA, TatB, and TatC proteins [111]. So far, only mitochondrial TatB and TatC proteins have been identified. Therefore, either the plant mitochondrial Tat pathway is highly unusual and functions without a TatA protein or TatA is yet to be identified. Second, why did plants retain a Tat pathway when it appears to have been lost in most other lineages (e.g., yeast, mammals, and some green algal species)? Are there more so-far unidentified substrates? Third, why do some species, such as *Chlamydomonas*, appear to lack both a Tat pathway and Bsc1? How is the Rieske Fe/S protein inserted in this situation? Are the Tat components from chloroplasts dual targeted to mitochondria or is there a completely different novel pathway for topogenesis of the Fe/S proteins in these organisms? The recent discovery that plant mitochondria contain a Tat pathway has opened up new and exciting areas of research and it will be fascinating to see how it develops in the future.

## 5. Case Study: A Comparison of Cox2 Biogenesis in Different Organisms

COX is the terminal enzyme in the respiratory electron transport chain. It catalyzes the transfer of electrons from soluble cytochrome *c* to molecular oxygen, coupled to protons getting pumped from the matrix to the IMS. The hydrophobic reaction center is made up of three mitochondrially-encoded subunits (Cox1, Cox2, and Cox3) and is surrounded by 11–13 nuclear-encoded subunits [112,113]. Post-assembly, COX forms a homodimer with cardiolipin as the connecting molecule, which is necessary for its activity and stability [114,115]. Electrons are transferred from the bivalent Cu_A_ site in Cox2 to heme *a* in Cox1 and then transferred intra-molecularly to the heme *a*_3_-Cu_B_ site where oxygen is bound [116]. Cox3 possibly modulates oxygen access or coordinates proton pumping. The nuclear-encoded subunits surrounding the core are required for assembly, stability, and dimerization of the enzyme, protection from the ROS, and regulation of the catalytic activity. Additionally, approx. 30 more nuclear-encoded ancillary factors are required for translational regulation, membrane integration, heme and copper insertion, and assembly of the various COX subunits [113]. Studies with yeast and human cell lines have identified several of these factors, most of which are conserved, but relatively very little information regarding their plant counterparts is currently available.

**Figure 3:**
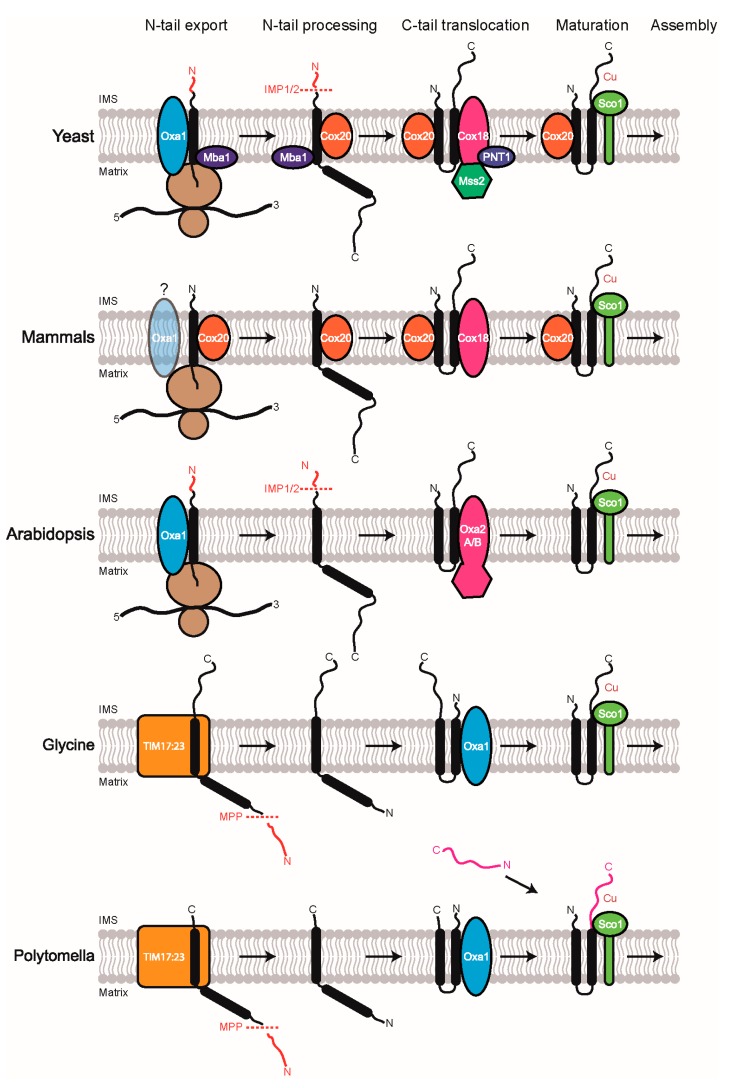
Cox2 biogenesis in yeast, mammals, *Arabidopsis*, *Glycine*, and *Polytomella*. Our current understanding of how Cox2 is inserted into the MIM of yeast (*Saccharomyces cerevisiae*) and mammals (*Homo sapiens*) is as depicted. The four steps of Cox2 assembly are N-tail export with TMH1 membrane insertion, N-tail processing, C-tail translocation with TMH2 membrane insertion and maturation by copper insertion. The last arrow represents the assembly step into complex IV. Based on this knowledge and some limited available studies with plant mitochondria, we have hypothesized the Cox2 insertion pathways for two plant species, *Arabidopsis thaliana*, *Glycine max*, and an algal species, *Polytomella* sp. In yeast, mammals, and *Arabidopsis*, Cox2 is synthesized by the mitochondrial ribosome whereas in *Glycine* and *Polytomella*, it is cytosolically synthesized and imported into the mitochondria. For full details, see the corresponding sections in the text.

Cox2 is an integral membrane protein containing two TMHs and both its N- and C-terminal regions are present in the IMS. The C-terminal region has a copper-binding Cx_3_C motif, which upon maturation forms the Cu_A_ site. Generally, the mitochondrial-encoded membrane proteins have small hydrophilic regions and especially the IMS-exposed stretches are very short, except for Cox2 that has a large IMS-exposed hydrophilic domain [97]. This exceptional feature of Cox2 demands more than one insertase machinery and additional factors for its biogenesis. Only after successful export of the N-terminus does the membrane potential-dependent process of the C-terminus export occur [117]. Also, while the majority of organisms encode Cox2 in the mitochondrial genome, some plant species have moved the gene encoding Cox2 to the nucleus. Thus, a comparison of how different organisms perform Cox2 biogenesis makes for an interesting undertaking as it highlights how much we currently know about the MIM protein insertion in yeast and humans and how much information we are lacking in reference to plant mitochondria (Figure 3).

### 5.1. Cox2 Biogenesis in Saccharomyces cerevisiae

Cox2 is the best studied membrane protein in yeast because the cleavage of its leader peptide serves as a simple indication for successful membrane insertion [76]. The *Cox2* gene has no introns and is transcribed by the general mitochondrial transcription machinery, followed by maturation with the help of 3′ processing machinery [113]. The translation requires a specific nuclear-encoded membrane-bound translational activator, Pet111 [118,119]. The coordination of Cox2 synthesis and the subsequent assembly is potentially regulated by the positively-charged N-terminal leader peptide and three additional downstream sequences [120,121]. Oxa1 interacts with the precursor of Cox2 (pCox2) and catalyzes the co-translational membrane insertion of the first TMH with the concomitant export of the N-terminal domain across the MIM into the IMS [117,122,123] (Figure 3). A specific chaperone called Cox20 interacts very early with pCox2 and makes it accessible to the membrane-bound IMP (inner membrane peptidase) complex for proteolytic processing. Cox20 also stabilizes and binds Cox2 until its assembly with the other subunits of COX [124] (Figure 3). Furthermore, the ribosome receptor Mba1 appears to stabilize the Cox20-ribosome complex and supports the transfer of Cox2 to the C-tail export module [125] (Figure 3). Then, Cox18, Mss2, and Pnt1 (pentamidine resistance factor) cooperatively facilitate the insertion of the second TMH with the simultaneous export of the C-tail [83] (Figure 3). While Pnt1 has no clear homologs in mammals and plants and appears to be a eukaryotic invention specific to fungi, Mss2 has a TPR domain resembling that of Tom70 [98,126], suggesting a prokaryotic origin. It has been suggested that Mss2 recognizes the Cox2 C-terminus in the matrix and promotes its translocation [98]. In the absence of Oxa1, Cox18, or Mss2, but not Pnt1, Cox2 proteins are rapidly degraded by proteases due to improper membrane insertion [127]. The last step in the biogenesis of Cox2 is its maturation by copper insertion at the CxExCGx_2_Hx_2_M motif in the β-barrel structure of the Cox2 C-tail, to form a divalent [Cu^2+^/Cu^1+^] complex called the Cu_A_ center [113]. Sco1 (suppressor of cytochrome oxidase deficiency 1) was identified as the metallochaperone and disulfide reductase which transfers copper to the Cu_A_ site in Cox2 [128,129,130,131] (Figure 3). Sco1 in turn receives copper from Cox17 [132,133]. It needs to be clarified whether the homolog of Sco1, Sco2, is also involved in the copper transfer. Although Sco2 deletion does not affect the biogenesis of COX, its overexpression partially rescues both Sco1 and Cox17 mutants [129]. After copper insertion, the mature Cox2 is ready to be incorporated into a COX assembly intermediate.

### 5.2. Cox2 Biogenesis in Homo sapiens

All thirteen mammalian mitochondrial-encoded genes lack introns and transcription is polycistronic with alternating structural genes and tRNAs. Processing of specific mRNAs is followed by polyadenylation and the mRNAs lack 5′ UTRs [113]. LRPPRC (leucine-rich PPR motif-containing protein) is the only known factor that stabilizes all mRNAs, including the COX core subunits [134]. The Cox2-specific translational activator in yeast, Pet111, is not conserved in human mitochondria [135]. It is still unclear how the mRNA translation in humans is activated. In contrast to the structure of yeast Cox2, the human Cox2 lacks a cleavable presequence. Oxa1L, the human homolog of Oxa1, is required for the biogenesis of complex I and V but not COX [81,136] (Figure 3). However, it can functionally complement the corresponding yeast mutant [137]. Hence, it is currently not clear whether Oxa1L actually performs the same role in Cox2 biogenesis as its yeast homolog. However, the role of Cox18 (Oxa2) appears to be conserved across species (Figure 3). During the insertion of its N-terminal TMH, Cox20 stabilizes Cox2 and subsequently Cox18 transiently interacts with Cox2 to promote the translocation of the Cox2 C-tail containing the apo-Cu_A_ site across the MIM (Figure 3). Then, the release of Cox18 from this complex coincides with the binding of the Sco1–Sco2–Coa6 (cytochrome oxidase assembly factor 6) copper metalation module to the Cox2–Cox20 intermediate [135,138] (Figure 3). Interestingly, although no homologs for Mss2 can be found in humans, the proteins corresponding to alternative splice variants of Cox18 [127] might perform a function similar to Mss2. Both Sco1 and Sco2 have a Cx_3_C copper-binding motif and are essential for COX assembly [139]. Moreover, Sco2 has an additional role in the synthesis of Cox2 [140], implying that it probably coordinates the synthesis and maturation processes of Cox2. It was proposed that following Cox2 metalation, Sco2 re-oxidizes Sco1, which makes them both ready for the next round of copper transfer. Thus, contrary to yeast Sco proteins, human Sco proteins have independent cooperative functions in the maturation of Cox2 or in COX assembly [141]. Cu(I) can be easily transferred from Cox17 to the two Sco proteins since they have a higher copper affinity [142]. Apart from their role in the biogenesis of Cox2, Sco1 and Sco2 are also involved in cellular copper homeostasis [143]. Very recently, a new protein called TMEM177 (transmembrane protein 177), which lacks a clear homolog in yeast, was found to form a complex with Cox2, Cox20, and the copper chaperones. Although its depletion or increased level affected Cox20 abundance and Cox2 stability, the assembly, abundance, and function of COX remained unaffected [144].

### 5.3. Cox2 Biogenesis in Arabidopsis thaliana

Very little previous research has been done to investigate Cox2 biogenesis in plants. Hence, the following speculations are mostly based on the observations made in yeast and humans, mentioned above. Based on sequence alignment, the *Arabidopsis* Cox2 is predicted to be synthesized as a precursor protein with a presequence, similar to the yeast Cox2. Plant Oxa1a can functionally complement a yeast *oxa1* deletion mutant [145]. Moreover, Oxa1a is essential for embryogenesis whereas homozygous deletion of Oxa1b does not affect the plant phenotype [84]. Therefore, it is most likely that Oxa1a mediates the co-translational insertion of the first TMH with concomitant export of the N-tail into the IMS (Figure 3). The presequence appears to be cleaved off by an IMP-like protease. Then, either Oxa2a or Oxa2b are possibly involved in the membrane insertion of the second TMH, accompanied by the C-tail export into the IMS (Figure 3). No homolog of Cox20 nor Mss2 nor Pnt1 has been identified in plants so far. However, it has to be noted that Mss2 and Pnt1 appear to be fungi-specific and are also not found in humans. As mentioned previously, it is interesting to speculate that the plant Oxa2 TPR domains and yeast Mss2 possibly play the same role in Cox2 biogenesis since the C-termini of Oxa2 proteins harbor a predicted TPR domain and the yeast Mss2 also has a predicted TPR domain. However, since there is no evidence that the TPR domains of Oxa2 proteins and Mss2 are related, convergent evolution is more likely. Perhaps, the TPR domain of Oxa2a/Oxa2b stabilizes the Cox2 C-terminus in the matrix until its export into the IMS by the insertase domain of Oxa2a/Oxa2b. Alternatively, it is also possible that similar to the TPR domain of Tom70 [86], the Oxa2 TPR domains interact with mtHsp70 or other chaperones that help in Cox2 stability and/or the assembly process, thus eliminating the need for a dedicated chaperone such as Cox20. After successful translocation, Cox2 can proceed for maturation by copper insertion most likely with the help of a protein similar to Sco1, called HCC1 (homolog of the yeast copper chaperone 1) (Figure 3). Interestingly, another Sco1 homolog, HCC2 does not affect COX activity but appears to be important for UV-B stress response [146,147]. *Arabidopsis* also has two genes that encode putative Cox17 homologs that are able to complement a yeast *cox17* null mutant [148,149,150].

### 5.4. Cox2 Biogenesis in Glycine max

The *Cox2* gene typically found in the mitochondrial genome of nearly all plants was very recently transferred to the nucleus in legumes. The nuclear-encoded Cox2 acquired a mitochondrial-targeting sequence bordered by two introns [151] and largely decreased the hydrophobicity of the first TMH [152]. The unusually long targeting signal of 136 amino acids consists of three parts that play different roles: the first 20 amino acids for mitochondrial targeting, the central portion for efficient import, and the last 12 amino acids derived from the mitochondrial-encoded protein are required for correct maturation of the imported protein. During import, the presequence is cleaved in a three-step process, independent of assembly [151] (Figure 3). Membrane insertion of the TMHs most likely involves TIM23 and Oxa1 before proceeding to the maturation step (Figure 3). Interestingly, when Cox2 is nuclear encoded, its C-terminus can be directly imported into IMS and will not require an export step from the matrix. Thus, the function of Oxa2 would not be required. However, the genome of *Glycine max* encodes for at least one Oxa2-like protein containing a similar TPR domain as the Oxa2 protein from *Arabidopsis*.

### 5.5. Cox2 Biogenesis in Chlorophycean algae

In contrast to the above case of legumes where the *Cox2* gene has been relocated to the nucleus as a single unit, in the chlorophycean algae *Chlamydomonas reinhardtii* and its colorless close relative *Polytomella* sp., the *Cox2* gene is split into complementary *Cox2a* and *Cox2b* genes before relocating independently to the nuclear genome and becoming lost from the mitochondrial genome. The *Cox2a* gene encodes the Cox2a protein that corresponds to the N-terminal half of the typical single polypeptide Cox2 and contains the two TMHs. The *Cox2b* gene encodes the Cox2b protein, which is equivalent to the C-terminal soluble IMS domain of the original protein. Both *Cox2a* and *Cox2b* are independently transcribed into mRNA and translated into separate polypeptides in the cytosol [153]. The mitochondria then import the two subunits and assemble them into the COX complex (Figure 3). Interestingly, in *Polytomella*, the two subunits are imported into mitochondria using different mechanisms. The Cox2a precursor exhibits a long cleavable mitochondrial-targeting sequence of 130 amino acids and appears to follow an energy-dependent import pathway involving mitochondrial-targeting sequence elimination by proteolytic processing and membrane integration of its two TMHs. The soluble Cox2b protein, lacking a cleavable targeting signal, is directly imported into the IMS via the TOM complex [154] (Figure 3). Researchers favor an import mechanism of Cox2a similar to the import route proposed for the soybean Cox2 precursor [151], described above. TIM23, after fully translocating the mitochondrial-targeting signal and the first TMH of Cox2a to the matrix, recognizes a stop transfer signal within the second TMH and thereby inserts it laterally into the MIM (Figure 3). Afterwards, the N-terminal part of the mature Cox2a would be translocated back in an export-like reaction by the Oxa1 machinery, in order to achieve the functional N-out C-out topology of Cox2a (Figure 3). It remains to be ascertained whether the mitochondrial disulfide relay through Mia40 and Erv1 is involved in the import of Cox2b. Probably after binding to the IMS-located Mia40 receptor, Cox2b interacts with the membrane-inserted Cox2a subunit to form the heterodimeric Cox2 subunit (Figure 3). Having derived from the process of split gene transfer, neither polypeptide has regions that are homologous to the known Cox2 proteins. While Cox2a has a 20 amino acid C-terminal extension, Cox2b has a 42 amino acid region exhibiting a high degree of charged amino acids at the N-terminus. According to the model proposed by Perez-Martinez et al., an interaction between the unique Cox2a C-terminal domain and the highly charged Cox2b N-terminal domain might stabilize the two Cox2 subunits in the COX complex [153]. However, it was recently shown that the C-terminal extension of Cox2b appears to be dispensable as it only weakly reinforces the Cox2a/Cox2b interaction. It was concluded that the hydrophilic domain of Cox2 is a highly stable structure that when split into Cox2a and Cox2b is able to maintain a strong interaction of the two fragments [155]. It is currently unknown whether the maturation step for the formation of the Cu_A_ center in Cox2b occurs before or after Cox2a/Cox2b interaction.

## 6. Outlook

While our understanding of the roles that plant mitochondria play in growth and development, stress responses and secondary metabolite production is ever increasing, our knowledge about how plant mitochondria insert and assemble their inner membrane proteins is based mainly on knowledge gained by research with yeast and mammalian mitochondria. Although many of the membrane protein insertion mechanisms identified in other organisms are also present in plant mitochondria, the fundamental differences with plant mitochondria—in containing double the normal number of Oxa protein homologs, having Oxa proteins with TPR domains, and also likely containing the most conserved of conservative sorting pathways, a Tat pathway—imply that the mechanisms of membrane insertion and assembly will also be different in plants. This necessitates specific studies on Oxa and Tat pathways in plant mitochondria to provide a better understanding of not only how plants insert and assemble the MIM proteins but also how these processes are regulated in different tissues and during various developmental growth stages. Plant mitochondria also appear to be missing several key proteins found in the mitochondria of other lineages, such as Cox20 or the components of the MITRAC (mitochondrial translation regulation assembly intermediate of COX) complex, which have been well studied in other organisms. This raises the possibility that plant mitochondria contain unique plant-specific proteins for performing these functions. Grouping all plants together is not a good idea because, considering the example of Cox2 biogenesis, there may be species-specific differences.
